# Effect of maternal height on caesarean section and neonatal mortality rates in sub-Saharan Africa: An analysis of 34 national datasets

**DOI:** 10.1371/journal.pone.0192167

**Published:** 2018-02-06

**Authors:** Esther Arendt, Neha S. Singh, Oona M. R. Campbell

**Affiliations:** 1 Department of Infectious Disease Epidemiology, London School of Hygiene and Tropical Medicine, London, United Kingdom; 2 Department of Global Health and Development, London School of Hygiene and Tropical Medicine, London, United Kingdom; The Hospital for Sick Children, CANADA

## Abstract

**Rationale:**

The lifecycle perspective reminds us that the roots of adult ill-health may start in-utero or in early childhood. Nutritional and infectious disease insults in early life, the critical first 1000 days, are associated with stunting in childhood, and subsequent short adult stature. There is limited or no opportunity for stunted children above 2 years of age to experience catch-up growth. Some previous research has shown short maternal height to lead to adverse birth outcomes. In this paper, we document the association between maternal height and caesarean section, and between maternal height and neonatal mortality in 34 sub-Saharan African countries. We also explore the appropriate height cut-offs to use. Our paper contributes arguments to support a focus on preventing non-communicable risk factors, namely early childhood under-nutrition, as part of the fight to reduce caesarean section rates and other adverse maternal and newborn health outcomes, particularly neonatal mortality. We focus on the Sub-Saharan Africa region because it carries the highest burden of maternal and neonatal ill-health.

**Methods:**

We used the most recent Demographic and Health Survey for 34 sub-Saharan African countries. The distribution of heights of women who had given birth in the 5 years before the survey was explored. We adopted the following cut-offs: Very Short (<145.0cm), Short (145.0–149.9cm), Short-average (150.0–154.9cm), Average (155.0–159.9cm), Average-tall (160.0–169.9cm) and Tall (≥170.0cm). Multivariate logistic regression was used to assess the contribution of maternal stature to the odds ratio of caesarean section delivery, adjusting for other exposures, such as age at index birth, residence, maternal BMI, maternal education, wealth index quintile, previous caesarean section, multiple birth, birth order and country of survey. We also look at its contribution to neonatal mortality adjusting for age at index birth, residence, maternal BMI, maternal education, wealth index quintile, multiple birth, birth order and country of survey.

**Results:**

There was a gradual increase in the rate of caesarean section with decreasing maternal height. Compared to women of Average height (155.0–159.9cm), taller women were protected. The adjusted odds ratio (aOR) for Tall women was 0.67 (95% CI:0.52–0.87) and for Average-tall women was 0.78 (95% CI:0.69–0.89). Compared to women of Average height, shorter women were at increased risk. The aOR for Short-average women was 1.19 (95% CI:1.03–1.37), for Short women was 2.06 (95% CI:1.71–2.48), and for Very Short women was 2.50 (95% CI:1.85–3.38). There was evidence that compared to Average height women, Very Short and Short women had increased odds of experiencing a neonatal death aOR = 1.95 (95% CI 1.17–3.25) and aOR = 1.66 (95% CI 1.20–2.28) respectively. When we focused on the period of highest risk, the day of delivery and first postnatal day, these aORs increased to 2.36 (95% CI 1.57–3.55) and 2.34 (95% CI 1.19–4.60) respectively. The aORs for the first week of life (early neonatal mortality) were 1.90 (95% CI 1.07–3.36) and 1.83 (95% CI 1.30–2.59) respectively.

**Conclusions:**

Short stature is associated with an increased prevalence of caesarean section and neonatal mortality, particularly on the newborn’s first days. These results are even more striking because we know that caesarean section rates tend to be higher among wealthier and more educated women, who are often taller and that the same patterns may hold for neonatal survival; in such cases, adjusting for wealth, education and urban residence would attenuate these associations. Caesarean sections can be lifesaving operations; however, they cost the health system and families more, and are associated with worse health outcomes. We suggest that our findings be used to argue for policies targeting stunting in infant girls and potential catch-up growth in adolescence and early adulthood, aiming to increase their adult height and thus decrease their subsequent risk of experiencing caesarean section and adverse birth outcomes.

## Introduction

The lifecycle perspective reminds us that the roots of adult ill-health can start in-utero or in early childhood [1, 2]. Nutritional, infectious disease, and other poverty related insults in early life, the critical first 1000 days, are associated with stunting in childhood, and subsequent short adult stature. The first 1000 days are also the critical time period for attaining one’s full adult height potential [3], with limited or no opportunity for stunted children above 2 years of age to experience catch-up growth.

Consistent evidence shows adult height is a good indicator of population health [4], and that the association between short stature and adverse mortality and morbidity outcomes persists even after adjusting for education, occupation and income [4]. An analysis of the Demographic and Health Surveys (DHS) for 54 low- and middle-income countries found that environmental differences dominate over genetic factors in determining average adult height [5], except for the Pygmy populations of Africa [6], suggesting that height is amenable to intervention. However over the last century, sub-Saharan Africa [4] has experienced the smallest gains, and in some cases even losses, in average adult height [5].

Some previous research has shown short maternal height leads to adverse obstetric, neonatal and foetal outcomes[7, 8]. A number of studies in developed and developing country settings support an inverse association between maternal height and caesarean section rate [9–15]. Furthermore, children of short women experience higher under-five mortality than those of average height and tall women [16, 17]. This association between maternal height and child mortality is most significant for the neonatal period as found using data from 109 DHS [17]. As far as we are aware, no study has explored the association between maternal height and neonatal mortality by age of death within the neonatal period. A number of studies point to an association between short maternal stature and preterm birth, intra-uterine growth restriction, low Apgar scores, and birth injuries such as clavicle fracture and brachial plexus injury [12, 18–20]. Finally, short maternal height is associated with stillbirths in a number of studies [18, 21, 22]. However, it is important to note that the studies were of varying quality, and one did not consider any potential confounding factors [10].

In this paper, we seek to document the association between maternal height and caesarean section, and between maternal height and neonatal mortality, in 34 sub-Saharan African countries. Our aim is to contribute to arguments to prevent non-communicable risk factors, namely early childhood under-nutrition, as part of the effort to reduce caesarean section rates and other adverse maternal and newborn health outcomes, particularly neonatal mortality. We anticipate that our evidence will help make stronger arguments for policy changes that improve nutrition and care for girls. We focus on Sub-Saharan Africa because the region carries the highest burden of maternal and neonatal ill-health. [23, 24].

## Methods

### Materials

**Fig 1:**
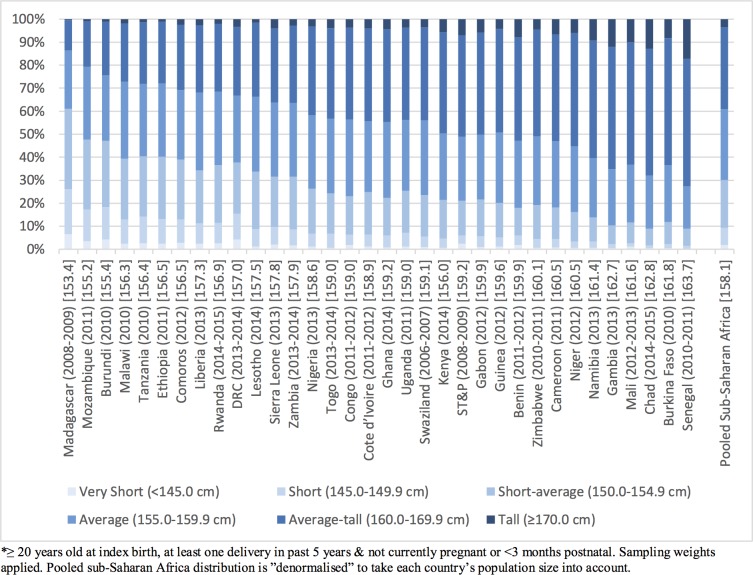
Proportion of women* by height categories in sub-Saharan Africa: Country (year of survey) [mean height eligible women].

We used the most recent Demographic and Health Survey (DHS) for the 34 Sub-Saharan African countries for which a survey was available in the period from 2006 to July 2016 (see Fig 1). Sub-Saharan Africa is made up of 49 countries, of which 34 (69%) are included in this study. The population of these countries make up over 80% of the total population of sub-Saharan Africa [25]. Furthermore, the surveys in each country comprised a large number of participating women, which has yielded a final sample size of over 100,000 women in each of our models. Having such a large sample, and it being representative of over 80% of the region increases confidence in the presented results. The DHS are nationally representative household surveys, which collect data based on standardized questionnaires. Women aged 15–49 are the main respondents, and there is a focus on information on births in the five years preceding the survey. Maternal height and weight are measured by the interviewers following a set procedure.

We examined the heights of women, aged 20 years and above, who had given birth in the 5 years before the survey. Women younger than 20 years-old at the most recent (index) birth in the five-year period were excluded as they may not have reached their full adult height at time of delivery. Although typically growth stops in girls shortly after menarche, so pregnant girls/women are typically no longer experiencing much growth, there is recent evidence to suggest that some catch-up growth might occur in adolescence and early adulthood[26]. Furthermore, women who were currently pregnant or who were less than 3 months postpartum were excluded, because we wished to adjust for body mass index (BMI) and were concerned it would be inaccurately measured in these periods. We adopted height cut-offs used by Özaltin and colleagues [17], but subdivided their last category to add a category of Tall. Our categories were thus: Very Short (<145.0cm), Short (145.0–149.9cm), Short-average (150.0–154.9cm), Average (155.0–159.9cm), Average-tall (160.0–169.9cm) and Tall (≥170.0cm).

### Analyses

All analyses were undertaken using STATA 14.1. Svyset commands were used with the subpop option, to account for clustering, weights and stratification. The weights for each survey were “denormalised” taking the country’s total population into account using the closest available estimates [25], ensuring estimates could be assigned to the Sub-Saharan Africa region. A dummy variable for country of survey was added to each model.

Multivariate logistic regression was used to assess the contribution of maternal stature to the odds ratio of caesarean section delivery, adjusting for other exposures[17, 27], such as age at the index birth (in 5-year groups), residence(urban/rural), maternal BMI(underweight, optimal, overweight, obese 1–3), maternal education (no education, primary only, secondary or higher), wealth index quintile, previous caesarean section (yes/no), multiple birth(yes/no), parity/birth order (first live birth, 2–3 previous live births, 4–5 previous live births, ≥6 previous live births), and country of survey. Because the DHS does not record the categories of caesarean section (elective vs. emergency), we could not differentiate by category of caesarean section in our analysis. We also examined the contribution of maternal stature to neonatal mortality adjusting for age at index birth, residence, maternal BMI, maternal education, wealth index quintile, multiple birth, birth order and country of survey.

### Ethical approval

Ethical approval was obtained from the London School of Hygiene and Tropical Medicine ethics committee on May 18^th^ 2016. DHS obtained the required local ethical approval and permission for each survey [28].

## Results

In total, 129,149 women were eligible for analysis; the 34 Sub-Saharan African DHS ranged in size from 1,074 women in Lesotho to 17,327 in Nigeria.

Fig 1 shows the distribution of heights by country, as well as the mean height. There was a ten centimetre difference in mean height between the tallest and shortest country in Sub-Saharan Africa. Madagascar had the shortest women, while Senegal had the tallest. In the pooled sample, 1.8% of women were categorised as Very Short (<145.0 cm), 7.5% as Short (145.0–149.9 cm), 21.0% as Short-average (150.0–154.9 cm), 30.7% as Average (155.0–159.9 cm), 35.5% as Average-tall (160.0–169.9 cm) and 3.5% as Tall (≥170.0 cm).

**Fig 2:**
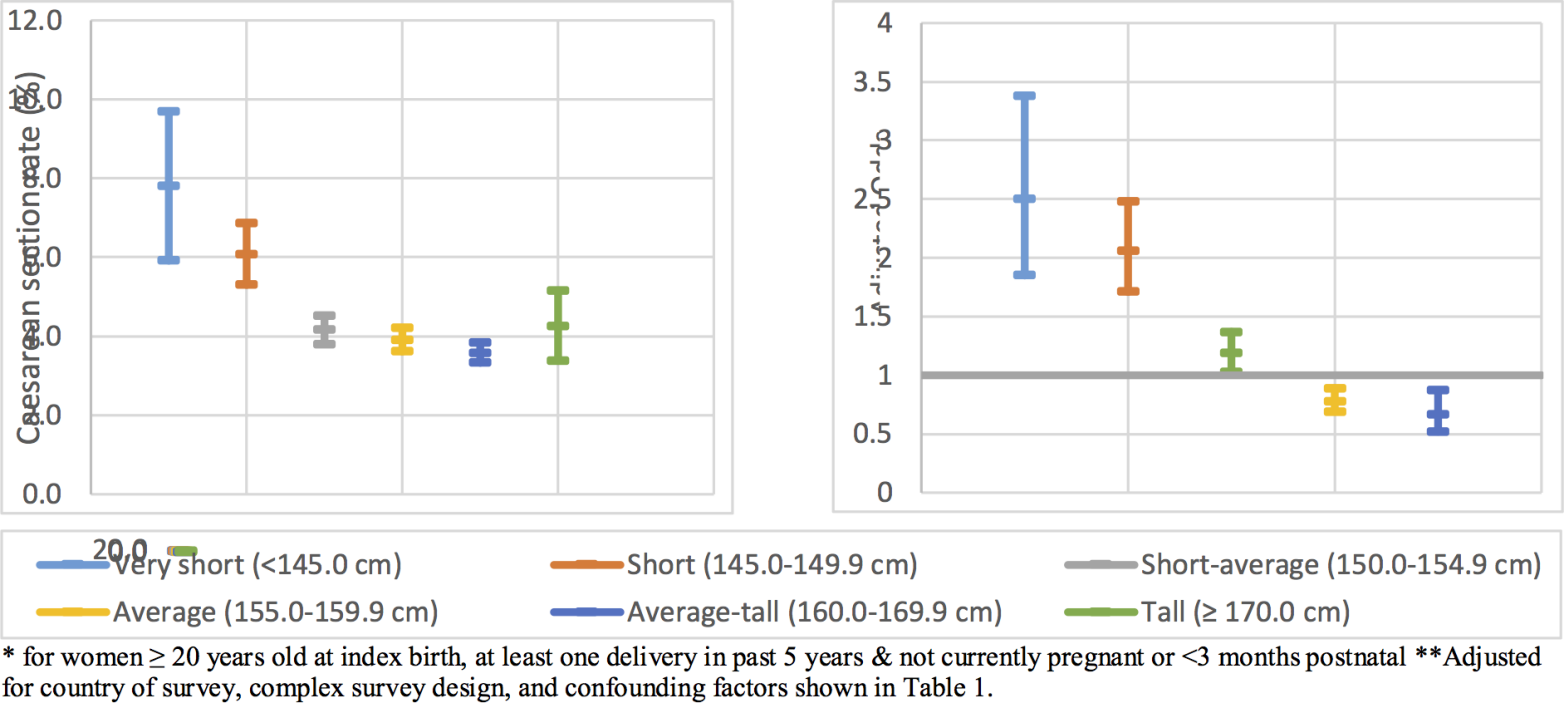
Caesarean section rate* by height categories in sub-Saharan Africa & adjusted odds ratios** of having a caesarean section compared to Average height women by height category in sub-Saharan Africa.

Table 1 and Fig 2 shows the crude and adjusted odds ratios of caesarean-section. There was a gradual increase in the odds ratio of caesarean section with decreasing maternal height. Compared to women of Average height (155.0–159.9cm), taller women were protected from having a caesarean section. The adjusted odds ratio (aOR) for Tall women was 0.67 (95% CI:0.52–0.87) and for Average-tall women was 0.78 (95% CI:0.69–0.89). Compared to women of Average height, shorter women were at increased risk. The aOR for Short-average women was 1.19 (95% CI:1.03–1.37), for Short women was 2.06 (95% CI:1.71–2.48), and for Very short women was 2.50 (95% CI:1.85–3.38).

**Table 1:** Unadjusted and adjusted odds ratios of caesarean section rates in Sub-Saharan Africa.

			Distribution (%) (n = 104,907)*	OR**	95% CI	P-value		Adjusted OR***	95% CI	P-value
Maternal height in cm										
	Very short <145.0		1.9%	2.27	(1.69–3.05)	<0.001		2.50	(1.85–3.38)	<0.001
	Short 145.0–149.9		7.5%	1.71	(1.44–2.03)	<0.001		2.06	(1.71–2.48)	<0.001
	Short-average 150.0–154.9		21.0%	1.09	(0.96–1.23)	0.194		1.19	(1.03–1.37)	0.015
	Average 155.0–159.9		30.5%	1.00				1.00		
	Average-tall 160.0–169.9		35.7%	0.90	(0.81–1.01)	0.086		0.78	(0.69–0.89)	<0.001
	Tall ≥170.0		3.5%	0.99	(0.75–1.30)	0.929		0.67	(0.52–0.87)	0.003
Previous Caesarean section										
	No		98.9%	1.00				1.00		
	Yes		1.1%	24.94	(21.08–29.50)	<0.001		28.97	(22.59–37.14)	<0.001
Age at birth (years)										
	20–24		28.2%	1.00				1.00		
	25–29		29.2%	1.09	(0.99–1.21)	0.068		1.52	(1.32–1.75)	<0.001
	30–34		21.0%	1.14	(1.03–1.26)	0.014		2.23	(1.89–2.63)	<0.001
	35–39		14.6%	1.21	(1.08–1.37)	0.001		3.40	(2.79–4.14)	<0.001
	40–44		5.9%	0.88	(0.75–1.04)	0.122		3.69	(2.80–4.84)	<0.001
	45–49		1.1%	0.68	(0.46–1.00)	0.052		2.82	(1.56–5.11)	0.001
Residence										
	Rural		69.3%	1.00				1.00		
	Urban		30.7%	3.25	(2.96–3.58)	<0.001		1.31	(1.15–1.49)	<0.001
Maternal BMI kg)/height(m)^2^										
	Underweight <18.5		11.5%	0.65	(0.52–0.79)	<0.001		0.79	(0.65–0.97)	0.022
	Optimal 18.5–24.9		67.0%	1.00				1.00		
	Overweight 25–29.9		15.5%	2.28	(2.01–2.59)	<0.001		1.54	(1.34–1.76)	<0.001
	Obese 1 30–34.9		4.4%	4.04	(3.45–4.74)	<0.001		2.47	(2.07–2.96)	<0.001
	Obese 2 35–39.9		1.2%	4.74	(3.59–6.26)	<0.001		2.43	(1.78–3.30)	<0.001
	Obese 3 ≥40		0.5%	6.24	(3.95–9.83)	<0.001		3.48	(2.17–5.58)	<0.001
Maternal education										
	No education		40.8%	1.00				1.00		
	Primary only		34.2%	2.16	(1.87–2.47)	<0.001		1.49	(1.27–1.75)	<0.001
	Secondary or higher		25.0%	5.07	(4.49–5.74)	<0.001		1.72	(1.45–2.05)	<0.001
Wealth index quintile										
	Poorest		20.7%	1.00				1.00		
	Poorer		20.6%	1.43	(1.23–1.67)	<0.001		1.17	(0.97–1.42)	0.100
	Middle		19.7%	2.22	(1.92–2.56)	<0.001		1.73	(1.43–2.09)	<0.001
	Richer		19.6%	3.24	(2.83–3.70)	<0.001		1.80	(1.49–2.17)	<0.001
	Richest		19.5%	7.33	(6.43–8.35)	<0.001		2.65	(2.16–3.25)	<0.001
Multiple birth										
	No		97.8%	1.00				1.00		
	Yes		2.2%	2.92	(2.44–3.49)	<0.001		5.01	(3.97–6.32)	<0.001
Birth order										
	First birth		11.0%	2.02	(1.84–2.21)	<0.001		2.90	(2.53–3.31)	<0.001
	2–3 previous births		33.5%	1.00				1.00		
	4–5 previous births		26.8%	0.64	(0.57–0.71)	<0.001		0.57	(0.50–0.66)	<0.001
	≥6 previous births		29.0%	0.46	(0.41–0.52)	<0.001		0.36	(0.29–0.43)	<0.001

* women ≥ 20 years old at index birth, at least one delivery in past 5 years, not currently pregnant or <3 months postnatal.

** Adjusted for country of survey and complex survey design (clustering, weights & stratification).

*** Adjusted for country of survey, complex survey design, and all variables in model.

Overall neonatal mortality rate at index birth of eligible women was 21.5 per 1,000 live births (95% CI 20.2–22.9). For the index birth the lowest neonatal mortality rate for these women were noted in Rwanda and Congo at 10.1 (95% CI 6.4–13.8; 95% CI 5.3–15.0); and the highest rate was noted in the Sierra Leone survey at 29.7 (95% CI 23.0–36.4).

**Fig 3:**
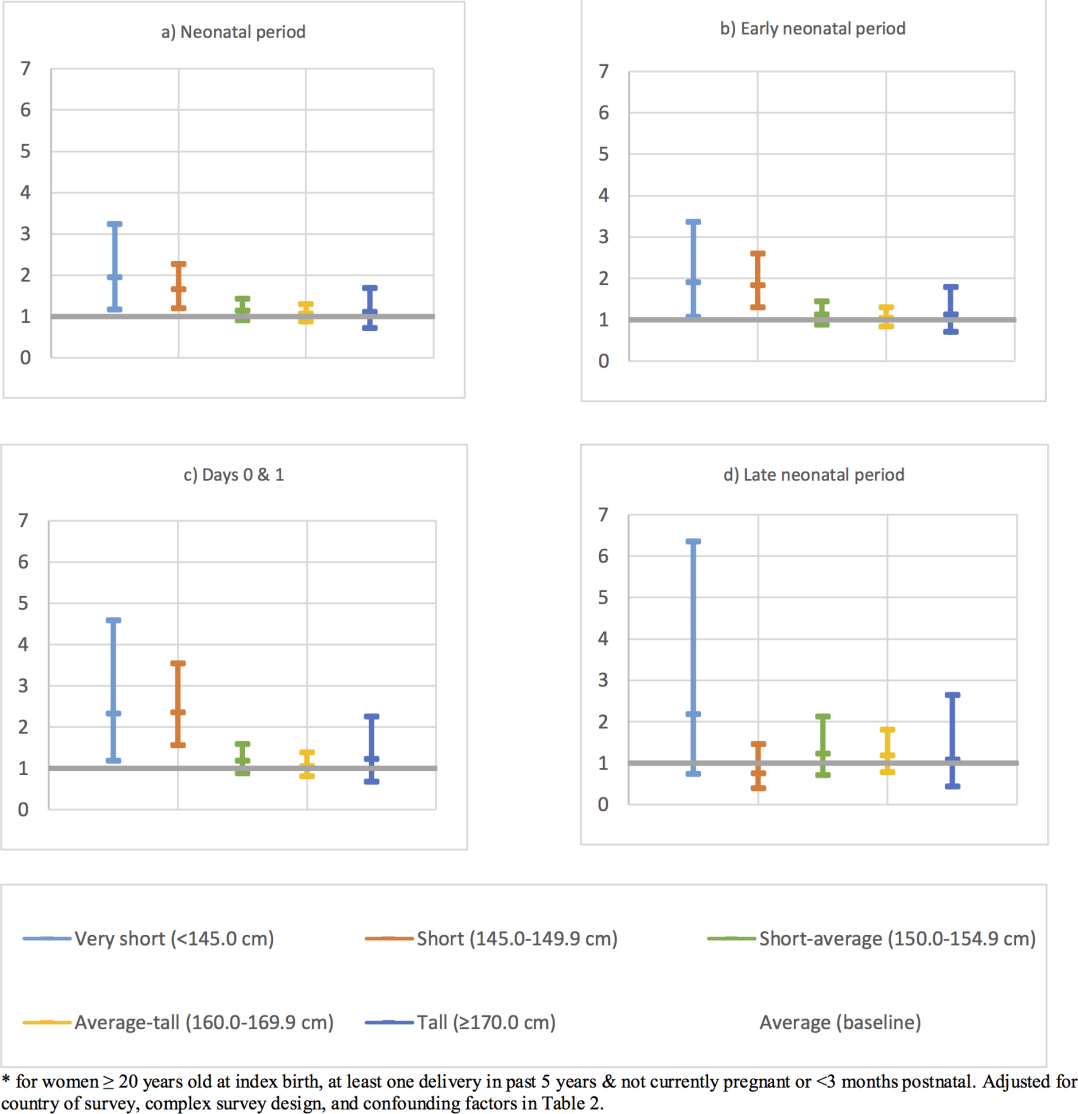
Adjusted odds ratios* of experiencing a neonatal loss compared to Average height women by height category for the a) neonatal period, b) early neonatal period, c) days 0 & 1 and d) late neonatal period.

Table 2 shows the shows the crude and adjusted odds ratios of neonatal mortality. The latter are also shown in Fig 3, as are the odds ratios for newborn mortality in day 0 and 1; the early neonatal period (days 0–6) and the late neonatal period (days 7–28). There was evidence that compared to Average height women, Very Short and Short women had increased odds of experiencing a neonatal death aOR = 1.95 (95% CI 1.17–3.25) and aOR = 1.66 (95% CI 1.20–2.28) respectively. When we focused on the period of highest risk, the day of delivery and first postnatal day, these aORs increased to 2.36 (95% CI 1.57–3.55) and 2.34 (95% CI 1.19–4.60) respectively. The aORs for the first week of life (early neonatal mortality) were 1.90 (95% CI 1.07–3.36) and 1.83 (95% CI 1.30–2.59) respectively.

**Table 2:** Unadjusted and adjusted odds ratios of neonatal mortality in Sub-Saharan Africa.

		Distribution (%) (n = 105,132*)	OR**	95% CI	P-value	Adjusted OR***	95% CI	P-value
Maternal height in cm								
	Very short <145.0	1.9%	1.86	(1.12–3.09)	0.017	1.95	(1.17–3.25)	0.010
	Short 145.0–149.9	7.5%	1.62	(1.18–2.23)	0.003	1.66	(1.20–2.28)	0.002
	Short-average 150.0–154.9	21.0%	1.10	(0.88–1.38)	0.402	1.14	(0.91–1.44)	0.246
	Average 155.0–159.9	30.5%	1.00			1.00		
	Average-tall 160.0–169.9	35.7%	1.10	(0.91–1.34)	0.316	1.07	(0.88–1.30)	0.503
	Tall ≥170.0	3.5%	1.23	(0.82–1.84)	0.310	1.12	(0.73–1.70)	0.608
Age at birth (years)								
	20–24	28.2%	1.00			1.00		
	25–29	29.2%	1.01	(0.83–1.22)	0.923	1.31	(1.02–1.71)	0.038
	30–34	21.0%	1.37	(1.14–1.65)	0.001	1.73	(1.28–2.35)	0.008
	35–39	14.6%	1.59	(1.32–1.93)	<0.001	2.03	(1.47–2.82)	<0.001
	40–44	5.9%	2.31	(1.84–2.91)	<0.001	2.97	(2.07–4.28)	<0.001
	45–49	1.1%	2.43	(1.51–3.91)	<0.001	3.34	(1.80–6.20)	<0.001
Residence								
	Rural	69.3%	1.00			1.00		
	Urban	30.75%	1.03	(0.90–1.19)	0.639	0.98	(0.80–1.20)	0.828
Maternal BMI kg)/height(m)^2^								
	Underweight <18.5	11.5%	1.06	(0.80–1.39)	0.691	1.09	(0.83–1.44)	0.525
	Optimal 18.5–24.9	67.0%	1.00			1.00		
	Overweight 25–29.9	15.5%	1.37	(1.10–1.70)	0.005	1.34	(1.07–1.67)	0.010
	Obese 1 30–34.9	4.4%	1.95	(1.41–2.69)	<0.001	1.93	(1.38–2.69)	<0.001
	Obese 2 35–39.9	1.2%	1.72	(1.00–2.95)	0.049	1.58	(0.90–2.76)	0.112
	Obese 3 ≥40	0.5%	1.56	(0.69–3.54)	0.290	1.25	(0.50–3.13)	0.636
Maternal education								
	No education	40.8%	1.08	(0.90–1.29)	0.433	1.13	(0.86–1.50)	0.374
	Primary only	34.2%	1.18	(0.99–1.40)	0.067	1.21	(0.94–1.55)	0.130
	Secondary or higher	25.0%	1.00			1.00		
Wealth index quintile								
	Poorest	20.7%	1.00			1.00		
	Poorer	20.6%	1.11	(0.91–1.36)	0.315	1.01	(0.78–1.31)	0.913
	Middle	19.7%	1.06	(0.86–1.29)	0.596	0.92	(0.71–1.18)	0.499
	Richer	19.6%	1.22	(1.00–1.48)	0.051	1.14	(0.87–1.49)	0.351
	Richest	19.5%	1.08	(0.88–1.33)	0.436	0.91	(0.67–1.25)	0.568
Multiple birth								
	No	97.8%	1.00			1.00		
	Yes	2.2%	6.21	(5.16–7.47)	<0.001	6.97	(5.47–8.88)	<0.001
Birth order								
	First birth	10.7%	1.78	(1.40–2.25)	<0.001	2.42	(1.79–3.26)	<0.001
	2–3 previous births	33.5%	1.00			1.00		
	4–5 previous births	26.8%	1.11	(0.93–1.33)	0.229	0.92	(0.72–1.18)	0.521
	≥6 previous births	29.0%	1.80	(1.54–1.7)	<0.001	1.04	(0.78–1.38)	0.810

* women ≥ 20 years old at index birth, at least one delivery in past 5 years, not currently pregnant or <3 months postnatal.

** Adjusted for country of survey and complex survey design (clustering, weights & stratification).

*** Adjusted for country of survey, complex survey design, and variables in model

## Discussion

In sub-Saharan Africa, we found that short stature in women was associated with increased caesarean section and increased neonatal mortality rates, particularly on the newborn’s first days of life (day 0 and 1). The unadjusted association of short stature with increased caesarean section was notable because we know that caesarean section rates tend to be higher among wealthier[29] and more educated[30] women, who are often taller; adjusting for wealth, education and urban residence increased the magnitude of the association observed between short stature and caesarean section.

To our knowledge, this is the first study to apply categorical height cut-offs to show the association between maternal height and caesarean section rates. It is also the first to apply these height categories and examine their association with neonatal mortality by age at death within the neonatal period (death on day 0 and 1, and in the early and late neonatal periods).

The distribution of height across the 34 countries showed substantial variation, and the mean height of countries differed by over 10 cm. The mean height for the region was not the shortest seen world-wide: the NCD Risk Factor Collaboration reported that in 2014, South Asian women were the world’s shortest [31]. However, over the last century, sub-Saharan Africa [4] has experienced the smallest gains, and in some cases even losses, in adult height [5].

Several studies previously reported an association between short stature and increased caesarean section [9–15], but most used binary height cut-offs. Our large sample size allowed us to adjust for numerous confounders, including socio-economic measures which are rarely collected in facility-based studies. While our main exposure of interest was height, we also found caesarean section was associated with having had a previous caesarean section (within the 5 years before the survey), having higher age, urban residence, higher BMI, higher education levels, higher wealth, multiple birth and having a first birth. These crude associations remained significant in our adjusted model. None of these associations were unexpected, except for the protective effect of being underweight (BMI < 18.5).

The general lack of access to caesarean section in much of Sub-Saharan Africa is pertinent to our findings. In1985, the WHO set a benchmark caesarean section rate of between 10% and 15% [32].Twenty eight of our 34 countries had caesarean section rates below 10%. Even Namibia, which had the highest caesarean section rate, had caesarean section rates below 10% in the poorest two quintiles. Rates below 10% suggest poor women cannot access medically indicated caesarean sections that could save their lives or those of their babies[33]. In the context of unequal and inadequate access to caesarean section, socioeconomic status negatively confounds the association between maternal height and caesarean section.

Very short and Short women in our study experienced more neonatal losses than Average height women. Özaltin et al., who studied 109 surveys of 54 countries found the same association. Both studies adjusted for mother’s age at birth and education, birth order, multiple birth status, household income, urban/rural residence, and country. We also adjusted for maternal BMI kg)/height(m)^2^ while they adjusted for preceding birth interval, era-born, and child’s sex. Table 3 compares the effect of maternal height on neonatal mortality in both studies, changing the reference category to be comparable[17]. Odds ratios approximate risk ratios when the prevalence of an outcome is <10% [34], which is the case for neonatal mortality. The general pattern is similar between the two studies, although we saw higher odds ratios for neonatal death among Very Short and Short women compared to Average height counterparts than were found by Özaltin and colleagues. This may be because they included women from a number of continents, and being Very Short or Short in Sub-Saharan Africa may have carried a higher risk for experiencing a neonatal loss than elsewhere.

**Table 3:** Comparison of our study findings on the effect of maternal height on neonatal mortality to those from a previous study.

		Our study (34 countries in sub-Saharan Africa)	Özaltin et al. (2010)* (54 countries globally)
		Adjusted OR	Adjusted RR
<145.0 cm		2.06	1.44
145.0–149.9 cm		1.61	1.22
150.0–154.9 cm		1.15	1.09
155.0–159.9 cm		1	1
160.0–169.9 cm	(one category in	1.01	0.91 (original baseline)
≥170.0 cm	Özaltin et al)	1.02	

*values adjusted to reflect baseline in ours study.

Our findings go beyond those of Özaltin and colleagues to show that the effect of height was most pronounced on neonatal mortality on day 0 and 1. When we divided the neonatal period into sub periods, the pattern of shorter women more neonatal deaths was not seen in the late neonatal period, but remained in the early neonatal period, and was most marked the day of delivery and first postnatal day (i.e. day 0 and 1). The fact that the association is most marked in the earliest neonatal period suggests a higher incidence of deaths due to intrapartum events, formerly termed birth asphyxia, in Very Short and Short women [35]. Our evidence on the association of short stature with higher caesarean section also supports this interpretation because caesarean section can indicate difficult labour.

The literature also suggests women of short stature have more stillbirths [18, 21, 22]. There are roughly as many stillbirths as neonatal deaths, but unfortunately the DHS do not collect stillbirth data in most countries [36]. We might expect that short stature would have led to more stillbirths, and that intrapartum stillbirths might have arisen through some of the main mechanisms that increased caesarean section rates and neonatal mortality on day 0 and 1. By missing stillbirths, we likely underappreciated the effect of short stature on the totality of adverse intrapartum events, and that the difference in odds ratios for caesarean sections by height categories might have d be more pronounced if all women requiring a caesarean section for foetal distress had access to one.

However, considering babies who are born to shorter women are more at risk of being born by caesarean section than those born to average height or tall women, we know these babies are also less likely to benefit from skin-to-skin and initiation of breastfeeding within the first hour of life[37], which are both protective actions for neonates[38].

### Strengths and limitations

The study included 34 of the 49 Sub-Saharan African countries, and represented over 80% of the total population of the region[25]. Furthermore, the surveys had high household and individual level response rates of 95% or greater[28] and the final sample size was large, at 129,149 women. Our main exposure of interest, height, was measured in a standardised manner, as was BMI.

On the other hand, our other variables relied on women’s self-report, and could have been affected by recall bias or heaping of ages at death. Moreover, information on caesarean section was limited to live births in the five years prior to the survey. Only women with two or more births in the five-year period would have had information on caesarean section prior to the index birth. Those whose previous births were outside this window would have been coded as not having a previous caesareans section, even if they had. Also the effect of multiple birth on the odds of mortality and caesarean section may have been underestimated because we are unsure how multiple births were coded in the DHS if they had discordant survival. For example, if one twin was a stillbirth and the other a live birth, they might have been recorded as twins, or the live born twin might have been misclassified as a singleton.

It would have been desirable to include gestational age at birth in the analysis, as this could be on the causal pathway between short maternal height and neonatal mortality. Shorter women have been shown to have higher odds of having preterm deliveries than their taller counterparts [39, 40]. Furthermore, data on the indication for caesarean section (elective vs emergency or Robson classification [41]) and the cause of neonatal death would have added to our understanding of the mechanisms for the association between maternal height and caesarean section and neonatal mortality however this information is not recorded in the DHS. Finally, a more extensive analysis of other adverse obstetric outcomes potentially associated with maternal height should be undertaken (e.g. obstetric fistula [42] or stillbirths [36].

The findings of increased caesarean section rates and neonatal deaths, in particular on day 0 and 1, indicates improved intrapartum care is required for short women. Rey et al. suggest using a height cut-off (<150cm) to screen women to refer to hospitals for childbirth [20]. However, most previous research found height had limited positive predictive value for adverse birth outcomes [12, 21, 43]. Furthermore, as per Rose’s prevention paradox, we know that that the majority of neonatal deaths will be born to non-short women, because they are the largest proportion of the population, even if at the individual level they face lower risks. For these reasons, we do not advise using maternal height as a screening tool to determine high-risk women who need higher-level maternity care.

Future research should explore if the observed trends from Sub-Saharan Africa remain true across other regions of the globe, and if the height cut-offs proposed here can be applied more generally. Our study excluded women less than 20 years of age as they may still be growing. Future research should also assess whether adolescent mothers were shorter than older women to provide evidence for the notion that their growth had not yet completed.

## Conclusions

Our study findings show that short stature is associated with adverse reproductive outcomes. Short maternal stature leads to more caesarean sections, and shorter women experience higher levels of neonatal loss. While caesarean sections can be lifesaving operations, they cost the health system and families more, and are associated with worse health outcomes.

The problems of short stature can be potentially mitigated by better intrapartum care, but are best addressed through nutrition and infection prevention outside the childbirth period. Our findings contribute to highlighting the importance of implementing policies and programmes promoting good health and nutrition of young girls and women throughout the life course. Adult height is primarily established by averting stunting during the first two years of a child’s life, i.e. the critical first 1000 days [4], but recent research has shown that some catch-up growth might occur in adolescence and early adulthood[26]. Policies should target improved infant nutrition and reduced infections in infancy, with the aim of increasing girls’ adult height and thus reducing future adverse birth outcomes, and targeting healthy diets for older children, adolescents and young adults to promote potential catch-up growth. About a fifth of stunting could be reduced in high burden countries with an annual investment of $9.6 billion to scale up ten evidence-based nutrition interventions [44], although all available interventions for nutrition and disease prevention are estimated to be able to avert only about a third of stunting in total in the 1000-day window [45]. Additional evidence suggests that addressing the intergenerational effects of undernutrition [45–47] are essential to reducing stunting, and underscores the importance of a life course approach to targeting high impact nutrition interventions on children, girls, and in pregnancy. Making gains in the adult height of females is also important since short maternal stature is associated with small-for-gestational-age births, which has in turn been linked to increased risk of neonatal and infant mortality, as well as long-term health consequences such as neurocognitive impairment and adult chronic disease [40, 48–51]. Policies targeting stunting in infant girls, that aim to increase their adult height and thus decrease their subsequent risk of experiencing caesarean section, adverse birth outcomes, and other aspects of poor child and adult health should be strongly supported.
